# A Rare Presentation of Bilateral Foot Drop in Anti-neutrophil Cytoplasmic Antibody (ANCA)-Negative Eosinophilic Granulomatosis With Polyangiitis (Churg-Strauss Syndrome): A Diagnostic Challenge

**DOI:** 10.7759/cureus.86765

**Published:** 2025-06-25

**Authors:** Muhammad Mohsin Isar, Mohammed Al-Banna, Mohammad Ghaith Hulo, Reem Abu Salah, Mahmoud Abughazal

**Affiliations:** 1 Acute Medicine, United Lincolnshire Teaching Hospitals NHS Trust, Boston, GBR; 2 Emergency Medicine, United Lincolnshire Teaching Hospitals NHS Trust, Boston, GBR; 3 Internal Medicine, United Lincolnshire Teaching Hospitals NHS Trust, Boston, GBR

**Keywords:** churg-strauss, eosinophilic granulomatosis with polyangiitis (egpa), foot drop, hyper-eosinophilia, purpuric rash

## Abstract

Eosinophilic granulomatosis with polyangiitis (EGPA), known as Churg-Strauss syndrome, is a rare multisystem vasculitis mainly affecting small- to medium-sized vessels. It is characterized by eosinophil-rich granulomatous inflammation and necrotizing vasculitis. EGPA is considered a part of the ANCA-associated vasculitis (AAVs), but it also shares features with hypereosinophilic syndrome (HES), reflecting the dual contributions of vascular inflammation and eosinophilic tissue infiltration to organ damage.

This case report describes a 65-year-old male presenting with progressive bilateral foot drop and marked eosinophilia, ultimately diagnosed with EGPA. His symptoms included sensorimotor deficits and purpuric skin lesions. Investigations revealed mononeuritis multiplex, significant eosinophilia, and pulmonary involvement. The patient was started on high-dose corticosteroids followed by cyclophosphamide, which led to marked clinical improvement.

This case underscores the importance of early recognition and management of EGPA, highlighting its varied clinical manifestations and the potential for favorable outcomes with appropriate management.

## Introduction

Eosinophilic granulomatosis with polyangiitis (EGPA) is a rare systemic vasculitis classified under the antineutrophil cytoplasmic antibody-associated vasculitis. The 1994 Chapel Hill Consensus Conference has defined EGPA as eosinophil-rich and granulomatous inflammation in a background of necrotizing vasculitis predominantly affecting the respiratory tract with involvement of small- to medium-sized vessels. It is highly associated with asthma and peripheral eosinophilia [1,2]. 

The incidence and prevalence of EGPA are approximately 1.7 and 14.25 cases per million persons [3]. This figure may be underestimated due to the challenges in diagnosis and the application of variable criteria in different series. The clinical presentations of EGPA are very varied since the condition is a multisystem disease. In addition to asthma, rhinosinusitis, and eosinophilia, considered hallmarks, patients may present with other variable organ involvement that complicates timely diagnosis [4]. 

Early recognition and accurate diagnosis of EGPA are critical for effective management, as delayed intervention can lead to significant morbidity. This case highlights the diagnostic complexity and variable clinical presentation of EGPA, emphasizing the importance of a multidisciplinary approach in recognizing this rare condition. 

We present the case of a 65-year-old male with a history of bronchial asthma who developed progressive bilateral lower limb weakness and ankle drop, ultimately diagnosed as EGPA. This case adds to the growing body of literature on the clinical variability and diagnostic challenges of EGPA. 

## Case presentation

A 65-year-old male farmer with a medical history of bronchial asthma, bronchiectasis, hiatus hernia, depression, and degenerative disc disease presented with progressive bilateral lower limb weakness and foot drop. The symptoms began eight weeks before presentation, following a fall that was preceded by transient weakness in his right leg and an inability to dorsiflex his right big toe. A week later, he experienced paresthesia over the dorsum and sole of the right foot, which progressed to a complete right foot drop. Within days, similar symptoms developed in the left leg, leading to left foot drop and confinement to a wheelchair two weeks before admission. His medications included carbocisteine, fluoxetine, diazepam, montelukast, pregabalin, and salbutamol inhalers.

On examination, petechial and purpuric rashes were observed on the medial thighs and dorsum of the right foot. The neurological assessment revealed intact cranial nerve function, with a complete absence of ankle dorsiflexion and big toe extension bilaterally. Plantar flexion was graded at 0/5 on the right and 1-2/5 on the left. Reflexes were brisk at the knees but absent at the ankles, with no plantar reflexes. Sensory loss to fine touch was noted bilaterally in the L5 and S1 dermatomes.

Routine blood tests showed leucocytosis and marked eosinophilia, prompting further investigation for systemic or inflammatory causes. Other tests, including C-reactive protein (CRP), erythrocyte sedimentation rate (ESR), glycosylated hemoglobin (HbA1c), thyroid function tests, vitamin B12, and folate levels, were within normal limits.

**Figure 1:**
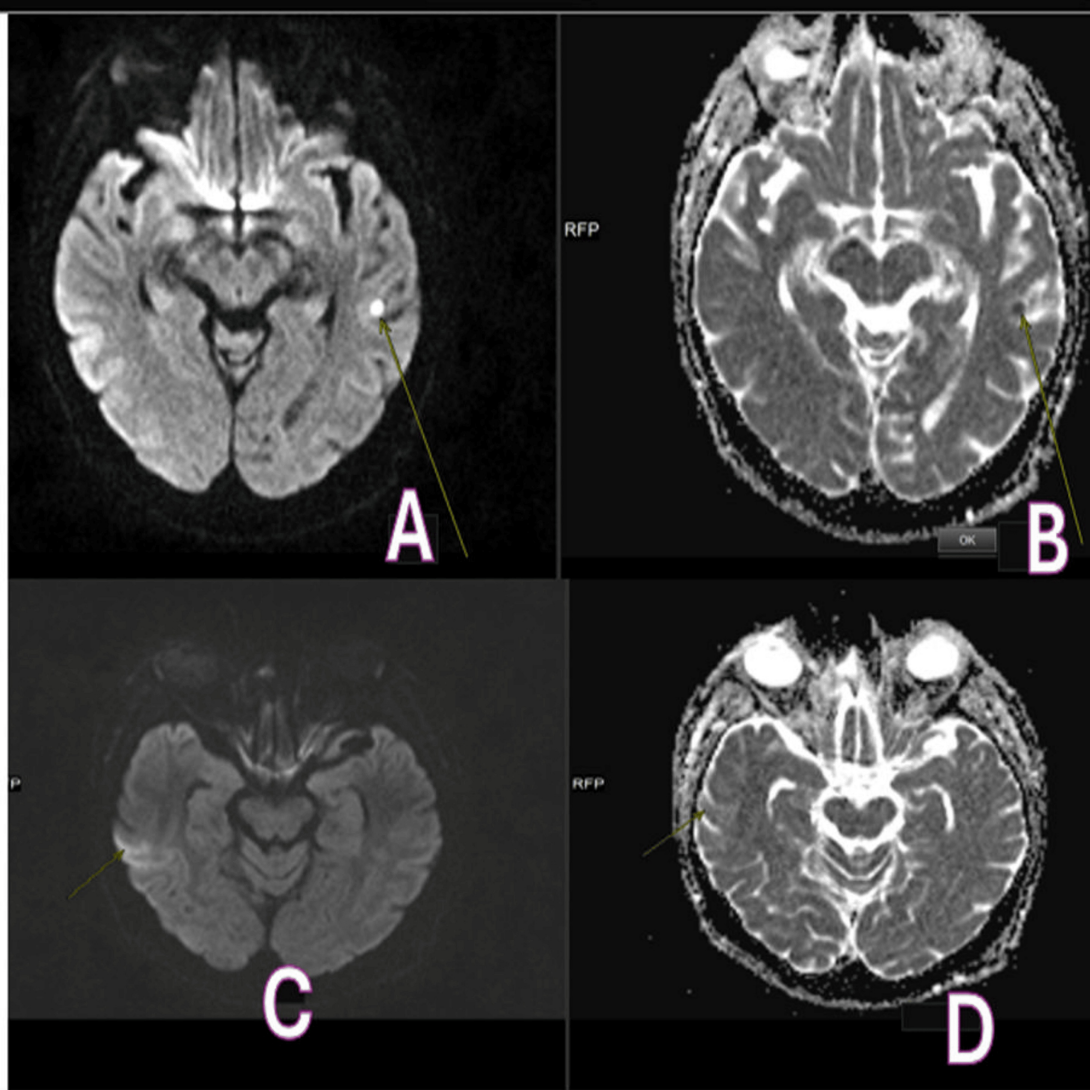
Head MRI with and without contrast. (A) A tiny focal hyperintensity (arrow) is seen on T1-weighted images, showing a blooming artefact on the gradient sequence in the left posterior capsulo-ganglionic region. (B) A tiny focal area of filling defect (arrow) is seen in the left posterior capsulo-ganglionic region. (C) A tiny focus of restricted diffusion (arrow) is seen involving the right temporal lobe, consistent with an acute lacunar infarct. (D) A tiny hyperintense focus (bright spot) (arrow) is seen involving the right temporal lobe, consistent with an acute lacunar infarct.

**Figure 2:**
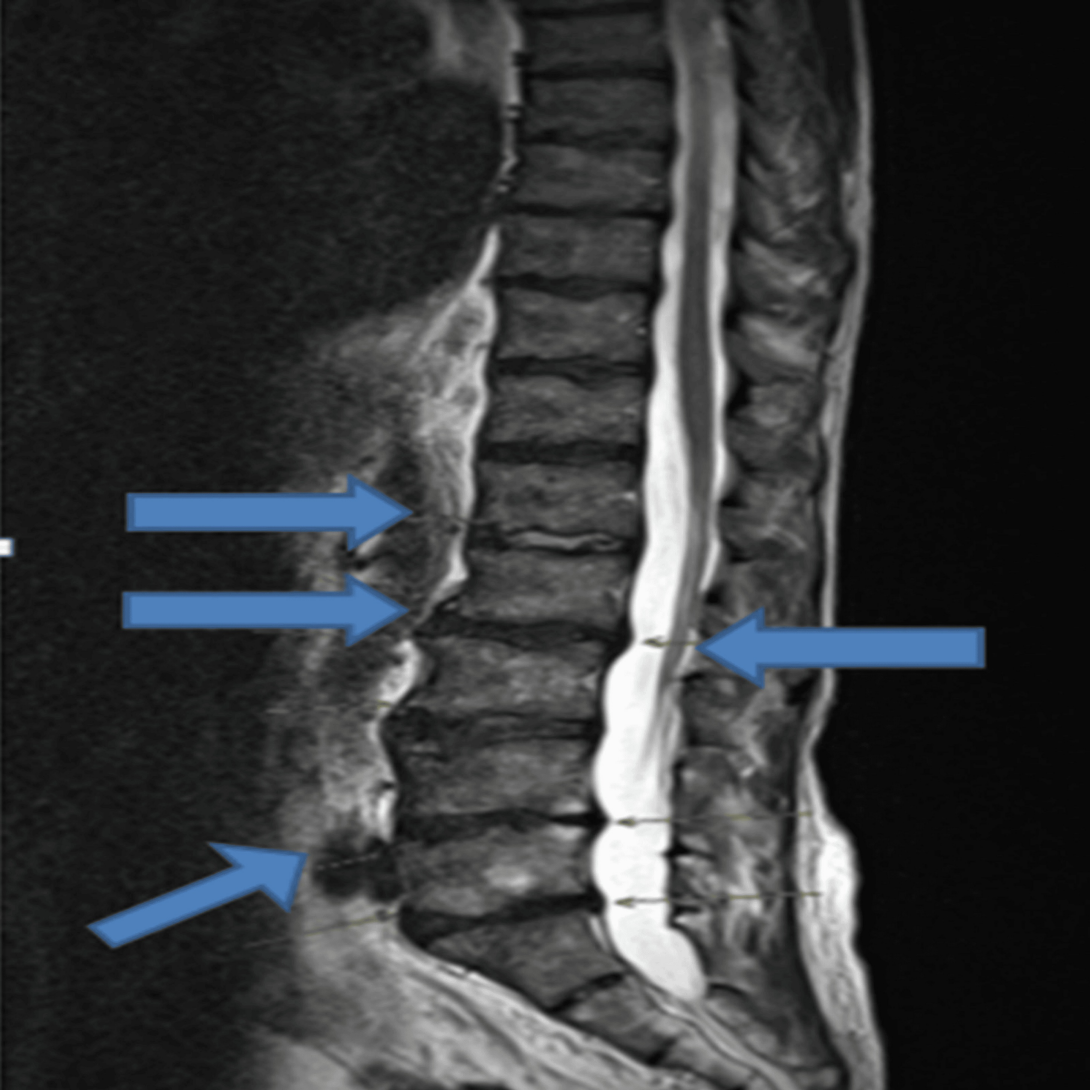
Whole spine MRI. Multiple blue arrows show stable multilevel spinal degeneration.

**Figure 3:**
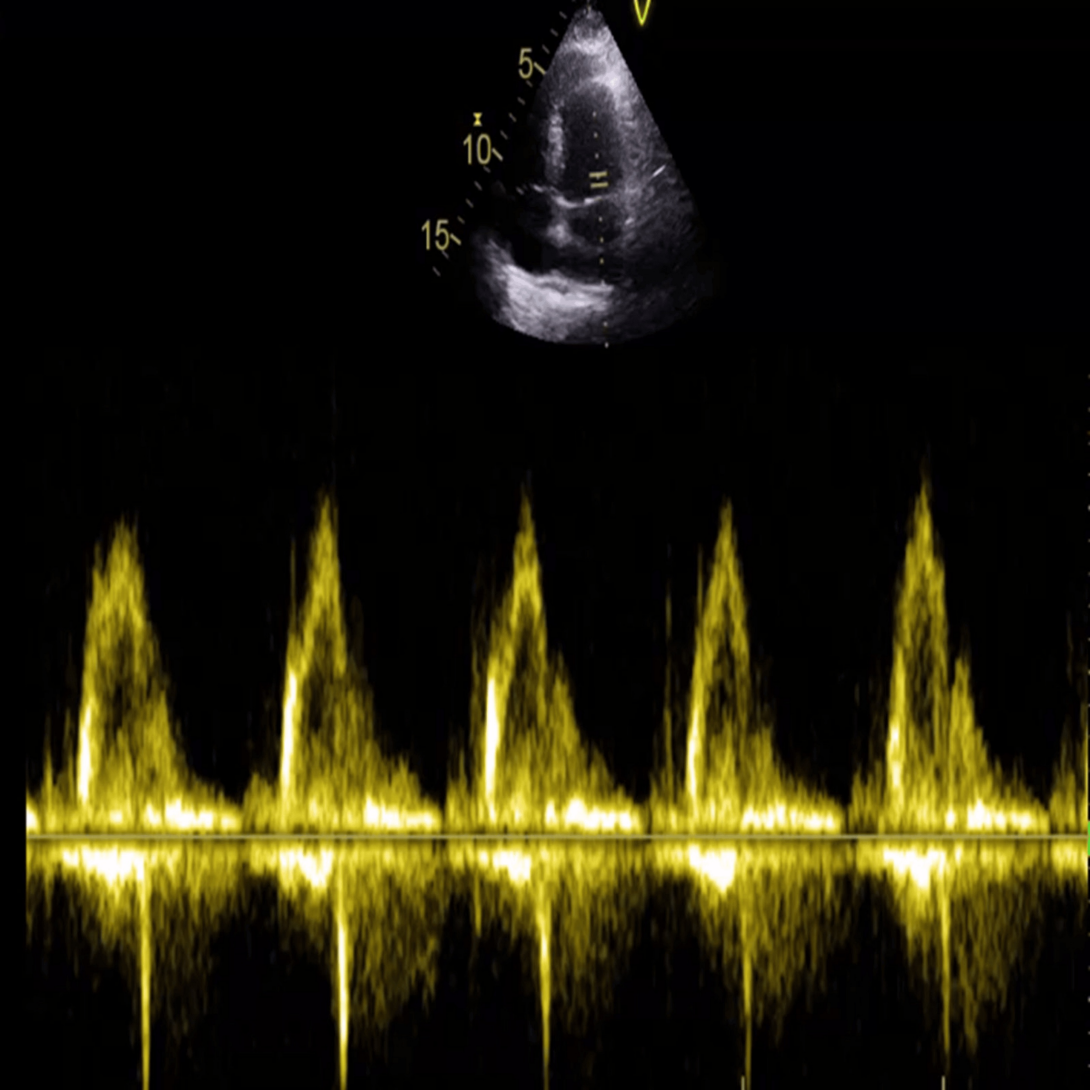
The echocardiogram showing well-defined heart chambers and properly functioning valves, with no evidence of structural abnormalities. The waveforms on Doppler imaging indicate normal blood flow and no signs of stenosis or regurgitation.

Following an initial normal CT of the head, an MRI of the head and whole spine was performed. Head MRI with contrast (Figure 1) revealed lacunar infarctions in the left posterior gangliocapsular region and the right temporoparietal region, while spinal MRI showed stable multilevel degenerative changes (Figure 2). The stroke team considered the lacunar infarctions to be incidental and likely of embolic origin, and suggested aspirin, echocardiography, and a follow-up CT head in three months. The echo did not show any vegetation or source of embolism (Figure 3).

**Figure 4:**
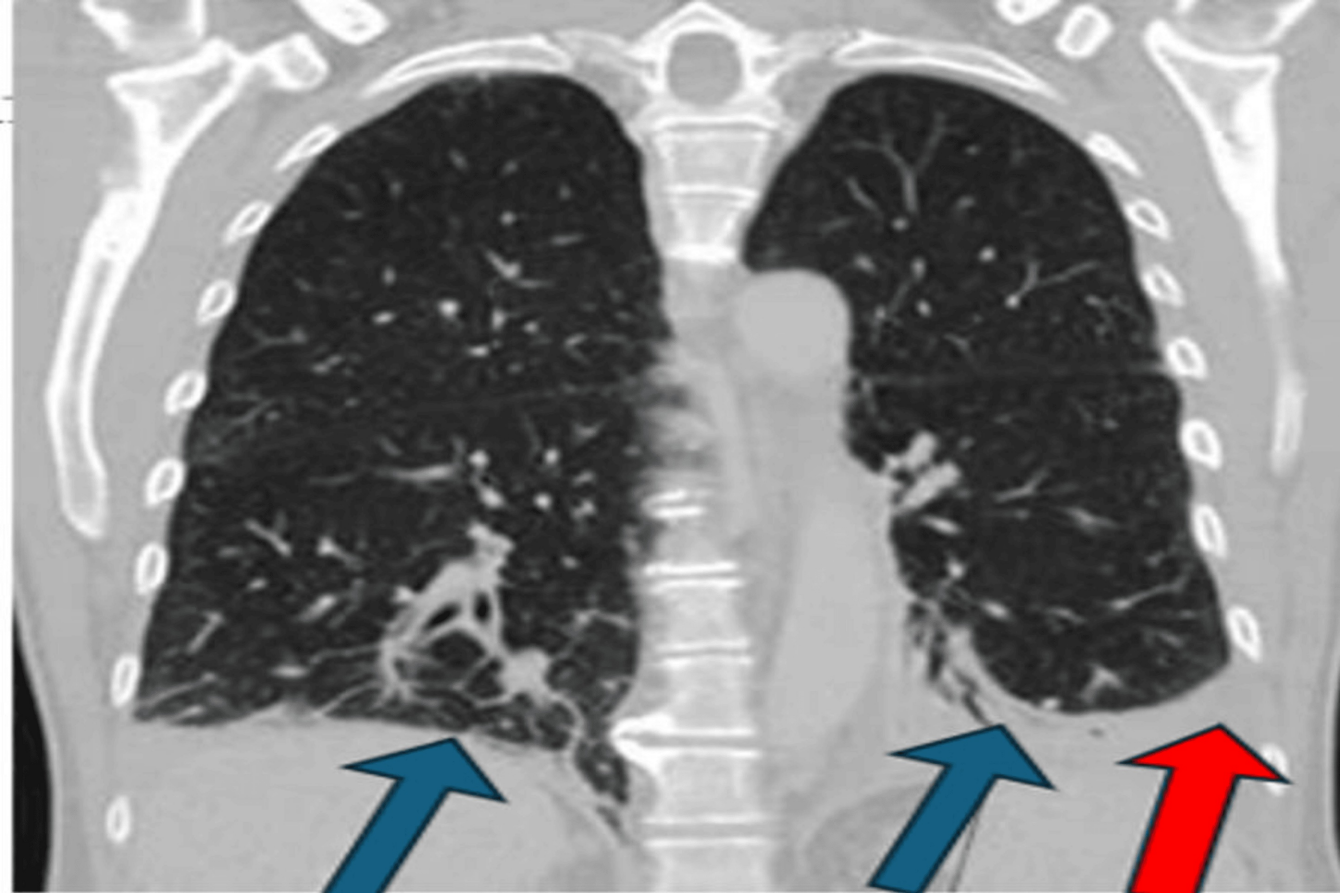
Chest CT scan reveals new-onset bibasal consolidation (blue arrows) and a mild left-sided pleural effusion (red arrow).

The significant eosinophilia and elevated white cell count raised concerns for alternative diagnoses such as paraneoplastic syndrome. As a result, a CT scan of the chest, abdomen, and pelvis (Figure 4) revealed bibasal consolidation, a mild left pleural effusion, and enlarged mediastinal and right hilar lymph nodes of an inflammatory nature. Hence, viral serology, including COVID-19, was sent, which all came negative. The requested anti-Hu and anti-Yo antibodies, as well as vascular endothelial growth factor levels, were negative, thereby excluding the possibilities of paraneoplastic syndrome and underlying malignancy, respectively. The peripheral blood film confirmed eosinophilia and showed no malignant cells.

These findings raised suspicion of vasculitis. As a result, an extensive diagnostic workup was performed, including viral PCR, autoimmune and vasculitis panels, such as antineutrophil cytoplasmic antibodies (ANCA) and antinuclear antibody (ANA), as well as hepatitis B and C and HIV serologies, complement (C3, C4) levels, cryoglobulin studies, and urinalysis. All results were unremarkable, as shown in Table 1.

**Table 1:** Blood results. ALT, alanine aminotransferase

Test name	Result	Reference range
Sodium	135 mmol/L	133-146
Potassium	4.2 mmol/L	3.5-5.5
Urea	4.2 mmol/L	2.5-7.8
Creatinine	77 umol/L	59-104
GFR	>90 mL/minute	
Glucose	6.5 mmol/L	3.0-6.0
ALT	11 U/L	0-41
C-reactive protein	7.4 mg/L	0-5
Hemoglobin	155 g/L	132-170
White cell count	20.5 × 10⁹/L	4.3-11.2
Eosinophil	13.74 × 10⁹/L	
Platelets	414	150-400
IgM	0.54 g/L	0.4-2.3
IgA	2.55 g/L	0.7-4.0
Serum Kappa	169.1 mg/L	3.3-19.4
Serum Lambda	96.0 mg/L	5.7-26.3
C3	1.94 g/L	0.90-1.8
C4	0.30 g/L	0.10-0.40
Neutrophil cytoplasm AB (ANCA)	Negative	
HIV screen	Negative	
Anti-nuclear antibody (ANA)	Negative	

**Figure 5:**
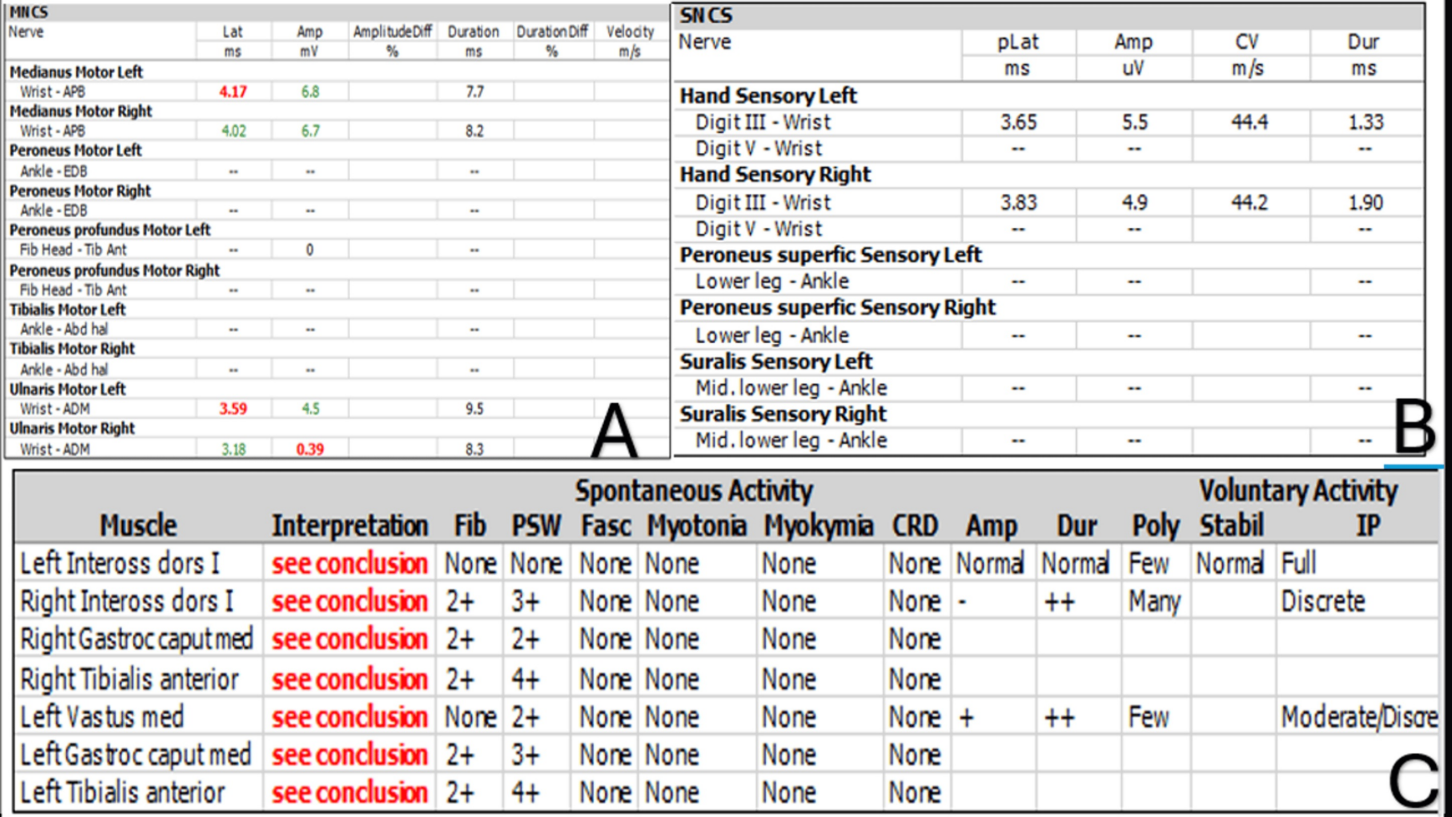
EMG/NCS study. (A) Motor studies performed in the lower limbs showed unrecordable responses. Ulnar motor responses were reduced. (B) Sensory responses in the lower limbs were unrecordable bilaterally. Ulnar sensory responses were also unrecordable. (C) Needle EMG studies showed marked active denervation. These findings indicate a very severe, asymmetric, sensory and motor axonal peripheral neuropathy affecting both upper and lower limbs. The pattern is consistent with acute, severe, and rapidly progressive mononeuritis multiplex. The abnormality now involves peripheral nerves in both lower limbs and the right ulnar nerve. EMG/NCS, electromyography and nerve conduction studies

**Figure 6:**
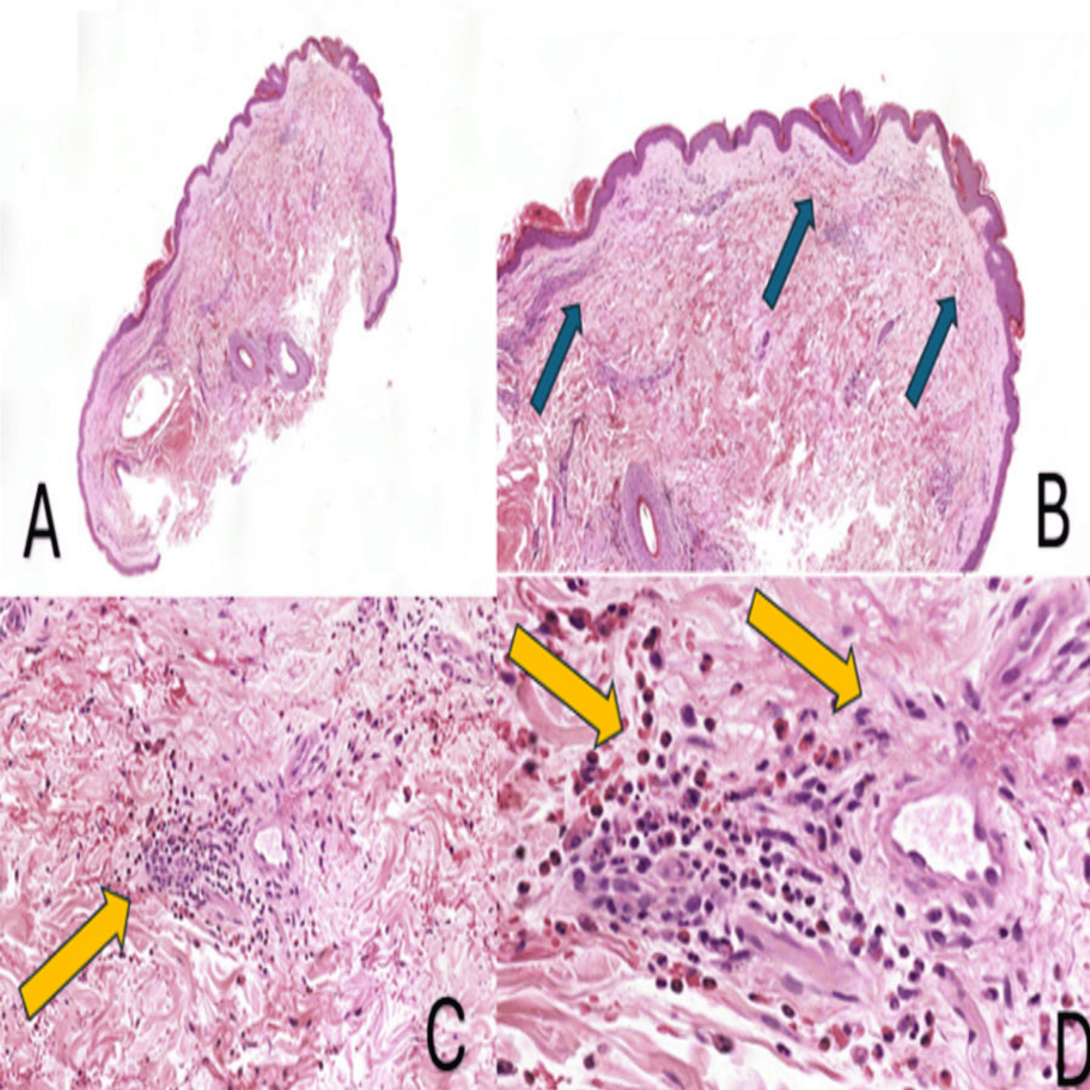
Skin biopsy. (A) Skin biopsy from the rash over the medial aspect of the left thigh. (B) (Blue arrows) Eosinophilic degranulation in the dermis. (C and D) (Yellow arrows) Superficial and deep eosinophilic infiltration in the interstitial and perivascular regions.

Electromyography and nerve conduction studies (EMG/NCS) confirmed acute, severe, and rapidly progressive mononeuritis multiplex, and Incisional skin biopsy from the rash over the medial aspect left thigh revealed superficial and deep eosinophilic infiltration and degranulation in the dermis, interstitium, and perivascular regions consistent with hyperoesinophilic syndrome (Figures 5-6).

The final diagnosis was ANCA-negative eosinophilic granulomatosis with polyangiitis (formerly called Churg-Strauss syndrome), based on multisystem involvement (neurological, respiratory, and dermatological), marked eosinophilia, a background of bronchial asthma, and biopsy findings.

Following the diagnostic workup, the rheumatology team was consulted and recommended initiating treatment with a three-day course of high-dose intravenous methylprednisolone, followed by 10 cycles of cyclophosphamide in combination with oral prednisolone. The patient demonstrated partial clinical improvement initially; however, a subsequent decline in renal function necessitated transitioning to azathioprine for maintenance therapy. Concurrently, antibiotics were administered to address suspected bilateral pneumonia identified on imaging. Throughout treatment, the patient showed significant clinical improvement, with motor strength in the lower extremities improving to 2-3/5 and partial recovery of motor function.

This case highlights the critical role of a systematic, multidisciplinary approach in diagnosing progressive neurological deficits associated with eosinophilia. The integration of clinical, imaging, histopathological, and electrophysiological findings was pivotal in identifying and managing this rare presentation of eosinophilic vasculitis with mononeuritis multiplex.

## Discussion

EGPA, also known as Churg-Strauss syndrome, is an uncommon multisystem vasculitis characterized by the presence of eosinophilic granulomatous inflammation, necrotizing vasculitis, and marked peripheral eosinophilia. Neurological involvement, especially peripheral neuropathy or mononeuritis multiplex, is one of the most common presentations and may account for 60%-70% of the cases. The patient's progressive bilateral foot drop and sensorimotor deficits are compatible with these typical neurological features [5]. 

EGPA is closely associated with asthma and eosinophilia. Asthma may precede the systemic manifestations by several years, and an eosinophilic phase, characterized by tissue infiltration, leads to impairment in multiple organ systems. In this patient, bibasilar consolidation and bilateral pleural effusion on chest CT are consistent with pulmonary involvement, which is commonly reported in EGPA. These manifestations highlight the systemic nature of the disease and underscore the importance of considering EGPA in cases of unexplained eosinophilia with multi-organ symptoms [1,6].

EGPA diagnosis is often a challenge because its features overlap with other eosinophilic disorders and vasculitis. The combination of mononeuritis multiplex, purpuric rash, hyper-eosinophilia, and pulmonary involvement in this case was very suggestive of EGPA. A negative ANCA does not rule out the diagnosis, as nearly 40% of cases are ANCA-negative. Skin biopsy findings of hypereosinophilic syndrome were important in providing histopathological evidence to support the diagnosis [4,7]. 

The 2022 diagnostic scoring system proposed by the American College of Rheumatology (ACR) and the European Alliance of Associations for Rheumatology (EULAR) assists in differentiating EGPA from other eosinophilic and vasculitic disorders. The components of this scoring system are presented in Table 2. 

**Table 2:** The 2022 diagnostic scoring system proposed by the American College of Rheumatology (ACR) and the European Alliance of Associations for Rheumatology (EULAR) assists in differentiating EGPA from other eosinophilic and vasculitic disorders. Source: [7]. EGPA, eosinophilic granulomatosis with polyangiitis

Obstructive airway disease	+3 Points
Nasal polyps	+ 3 Points
Mononeuritis multiplex points	+1 Points

Peripheral blood eosinophilia ≥1×10⁹/L	+5 Points
Biopsy showing eosinophilic vasculitis, perivascular eosinophilic infiltration, or eosinophil-rich inflammation	+ 2 Points
Cytoplasmic antineutrophil cytoplasmic antibody (ANCA) or anti-proteinase 3-ANCA positivity	-3 Points
Hematuria	-1 Point

A total score of ≥6 strongly supports the diagnosis of EGPA, with a sensitivity of 85% and a specificity of 99% for distinguishing EGPA from other eosinophilic disorders and types of vasculitis [8].

Applying the 2022 ACR-EULAR diagnostic scoring system to this patient yields a total score of 11, strongly supporting the diagnosis of EGPA. The patient fulfilled key criteria, including peripheral blood eosinophilia ≥1 × 10⁹/L (13.74 × 10⁹/L, 3 points), a history of asthma (3 points), peripheral neuropathy attributed to vasculitis (bilateral foot drop and mononeuritis multiplex, 2 points), pulmonary infiltrates not caused by infection (bibasilar consolidation and pleural effusion, 1 point), and biopsy findings of eosinophilic infiltration (skin biopsy consistent with hypereosinophilic syndrome, 2 points). There was no evidence of nasal polyps (0 points). The cumulative score of 11 meets the threshold for diagnosing EGPA with high sensitivity and specificity. These findings underscore the utility of the ACR-EULAR scoring system in accurately diagnosing EGPA in complex cases. 

The standard approach to the treatment of EGPA typically consists of high-dose corticosteroids as the first line of treatment, combined with the addition of other immunosuppressive agents, such as cyclophosphamide, in severe or refractory diseases. In this case, the patient’s significant improvement following methylprednisolone and cyclophosphamide underscores the efficacy of prompt immunosuppressive therapy. Timely diagnosis and treatment are cardinal in preventing irreversible complications, particularly neurological deficits. The muscle strength and functional recovery noted in this patient underscore the potential for full recovery with timely diagnosis and treatment [9,10]. 

This case highlights the importance of a multidisciplinary approach in managing EGPA. Collaboration between neurologists, rheumatologists, pulmonologists, and pathologists is essential to achieve accurate diagnosis and optimal care. Given the rarity and diverse presentations of EGPA, continued documentation of such cases contributes to the understanding of its clinical spectrum and guides management strategies [11]. 

## Conclusions

EGPA is a rare and complex multisystem vasculitis, often presenting with symptoms that may be variable, including neurological deficits and pulmonary involvement. This case highlights the importance of early diagnosis, guided by thorough clinical assessment and multidisciplinary teamwork.

Early initiation of corticosteroids combined with immunosuppressive therapy, as demonstrated in this case, led to significant improvement and helped prevent long-term complications. This underscores the need for greater awareness among clinicians.
